# In-Sn-Zn Oxide Nanocomposite Films with Enhanced Electrical Properties Deposited by High-Power Impulse Magnetron Sputtering

**DOI:** 10.3390/nano11082016

**Published:** 2021-08-06

**Authors:** Hui Sun, Zhi-Yue Li, Sheng-Chi Chen, Ming-Han Liao, Jian-Hong Gong, Zhamatuofu Bai, Wan-Xia Wang

**Affiliations:** 1 School of Space Science and Physics, Shandong University, Weihai 264209, China; huisun@sdu.edu.cn (H.S.); jamtuub@foxmail.com (Z.B.); 2 Department of Physics, The University of Hong Kong, Hong Kong 999077, China; lizhiyue007@hotmail.com; 3 Shenzhen Institute for Quantum Science and Engineering, Southern University of Science and Technology, Shenzhen 518055, China; 4 Department of Physics, Southern University of Science and Technology, Shenzhen 518055, China; 5 Department of Materials Engineering and Center for Plasma and Thin Film Technologies, Ming Chi University of Technology, Taipei 243, Taiwan; 6 College of Engineering and Center for Green Technology, Chang Gung University, Taoyuan 333, Taiwan; 7 Department of Mechanical Engineering, National Taiwan University, Taipei 106, Taiwan; mhliaoa@ntu.edu.tw; 8 School of Mechanical, Electrical and Information Engineering, Shandong University, Weihai 264200, China; gongjh@sdu.edu.cn (J.-H.G.); wang_wanxia@sdu.edu.cn (W.-X.W.)

**Keywords:** ITZO film, high-power impulse magnetron sputtering, duty cycle, pulse off-time, electrical properties

## Abstract

In-Sn-Zn oxide (ITZO) nanocomposite films have been investigated extensively as a potential material in thin-film transistors due to their good electrical properties. In this work, ITZO thin films were deposited on glass substrates by high-power impulse magnetron sputtering (HiPIMS) at room temperature. The influence of the duty cycle (pulse off-time) on the microstructures and electrical performance of the films was investigated. The results showed that ITZO thin films prepared by HiPIMS were dense and smooth compared to thin films prepared by direct-current magnetron sputtering (DCMS). With the pulse off-time increasing from 0 μs (DCMS) to 2000 μs, the films’ crystallinity enhanced. When the pulse off-time was longer than 1000 μs, In_2_O_3_ structure could be detected in the films. The films’ electrical resistivity reduced as the pulse off-time extended. Most notably, the optimal resistivity of as low as 4.07 × 10^−3^ Ω·cm could be achieved when the pulse off-time was 2000 μs. Its corresponding carrier mobility and carrier concentration were 12.88 cm^2^V^−1^s^−1^ and 1.25 × 10^20^ cm^−3^, respectively.

## 1. Introduction

Si-based thin-film transistors (TFTs) are widely used in liquid crystal displays, sensors, logic integrated circuits, etc. [1,2,3]. However, the high temperatures required for the formation of Si materials severely limits their applications in novel optoelectronic devices. For instance, flexible electrical devices and wearable devices are being developed rapidly nowadays but the flexible substrates used in these devices possess poor heat resistance, which makes Si-based TFTs unusable in these fields. Conversely, amorphous oxide semiconductors can be fabricated at room temperature [4,5,6]. Such materials combine good light transmittance and conductivity. Therefore, TFTs based on such materials are gradually replacing Si-based TFTs in certain areas. To date, the amorphous oxide semiconductor that has been most widely studied and has achieved commercial applications is In-Ga-Zn-O (IGZO) [7,8]. Its good uniformity and high carrier mobility (~10 cm^2^/Vs) have led to it attracting much attention in recent years [9]. Unfortunately, during the traditional back-channel-etching process used to manufacture amorphous IGZO TFTs, IGZO reacts easily with weak acids [10,11]. Furthermore, the field-effect mobility of IGZO TFT is still inadequate to drive high-frame-rate displays [12]. Therefore, it is necessary to explore other amorphous oxide semiconductors.

In-Sn-Zn-O (ITZO) is a novel transparent conductive material that replaces Ga_2_O_3_ in IGZO with more chemically stable SnO_2_, which helps to endow ITZO with better etching-resistance ability [13]. Meanwhile, the direct spatial overlap of the orbitals between Sn 5s orbital and In 5s can enhance the mobility of the electrons within the conduction band minimum, leading to a higher carrier mobility [14]. In addition, compared with the substitution of Zn^2+^ by trivalent Ga^3+^, the substitution of Zn^2+^ by tetravalent Sn^4+^ will release more free electrons and improve the electrical properties of the films [15]. As a result, ITZO-based TFTs have high potential for the development of next generation displays due to their good etching-resistance during the back-channel-etching process.

Currently, magnetron sputtering and the sol-gel method are most commonly used to prepare ZnO-based thin films [16,17,18]. In particular, magnetron sputtering has attracted much attention due to its low deposition temperature, fast sputtering speed, uniform film formation, and good repeatability [19,20,21]. However, the films deposited by traditional magnetron sputtering method present loose structure with many defects, greatly affecting the films’ performance [22]. The relatively recently developed high-power impulse magnetron sputtering (HiPIMS) technology has an important advantage in its high target ionization rate, which can improve the activity of the various species during the sputtering process [23,24,25,26]. In addition, due to the high instantaneous power density applied on the target, the energy of the incident species to the substrate is effectively increased, resulting in the formation of a denser and more uniform film, thereby reducing the carrier scattering and enhancing the carrier mobility [27,28]. To the best of our knowledge, no other groups have prepared ITZO films using HiPIMS technology. In the current work, the optoelectronic properties of ITZO films prepared by HiPIMS technology under different duty cycles were investigated.

## 2. Experimental Details

ITZO thin films with a thickness of 100 nm were deposited through HiPIMS technology on glass and silicon substrates from an ITZO target (99.9% purity, In_2_O_3_:SnO_2_:ZnO = 30:35:35 at.%, Φ = 76.2 mm) at room temperature. The working pressure was 0.7 Pa with the Ar flow rate maintained at 20 sccm. The sputtering power of the HiPIMS power supply was 300 W, while the pulse on-time (*t*_on_) remained at 50 μs. The pulse off-time (*t*_off_) varied from 0 to 2000 μs during the deposition process. The duty cycle is defined as the ratio between the *t*_on_ and the sum of *t*_on_ plus *t*_off_, and therefore reduces with an increase in *t*_off_. The deposition parameters maintained during the deposition are summarized in Table 1.

The sputtering voltage and current variation of HiPIMS power output were monitored by oscilloscope (Rigol DS5202CA, Rigol Technologies. Inc., Beijing, China). The films’ thickness was detected by step profiler (Kosaka Surfcoder, Kosaka Laboratory Ltd., Tokyo, Japan). The films’ composition was characterized by electron probe *X*-ray microanalyzer (EPMA, JEOL JXA-8200, JEOL, Tokyo, Japan). The structural properties of ITZO films were analyzed through *X*-ray diffractometer (XRD, Rigaku Ultima IV, Tokyo, Japan). The surface roughness of the films was measured by atomic force microscope (AFM, DI-Dimension 3100, Digital instruments, Bresso, Italy). The microstructure of the specimens prepared by focused ion beam (FIB) milling was observed on cross-sections by high resolution transmission electron microscopy (HR-TEM, JEOL JEM-2100, JEOL, Tokyo, Japan). The films’ electrical properties were obtained by the Hall effect measurement system (AHM-800B, Agilent Technologies, Santa Clare, CA, USA).

## 3. Results

**Figure 1:**
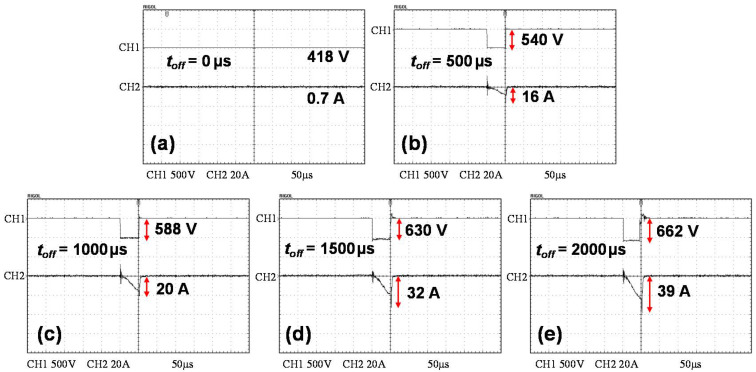
The sputtering voltage and current with different sputtering pulse off-times (*t*_off_): (**a**) 0 μs, (**b**) 500 μs, (**c**) 1000 μs, (**d**) 1500 μs, and (**e**) 2000 μs.

**Figure 2:**
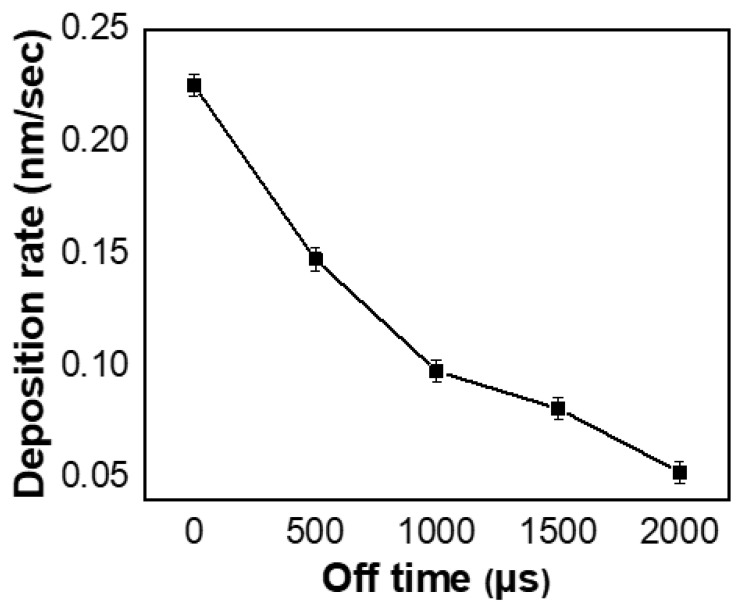
Deposition rate at different pulse off-times.

Figure 1 shows the variation of the sputtering voltage and current on the target with the pulse off-time prolongation during the deposition process. Both of them increased with the extension of the pulse off-time. Consequently, the peak power density on the target also rises gradually. The variation of the duty cycle and the calculated peak power density as a function of *t*_off_ are given in Table 2. As the pulse off-time extended from 0 μs to 2000 μs, the target peak power density rises greatly from 6.42 to 531.97 W·cm^−2^. However, the deposition rate monotonically decreased with the extension of pulse off-time (Figure 2). With increasing pulse off-time, the reduction in the effective sputtering period resulted in fewer target atoms being sputtered, which in turn reduced the deposition rate.

Table 3 shows the relationship between the pulse off-time and the film’s composition. The content of In, Sn, Zn, and O in the film changed slightly as the pulse off-time was extended. When the pulse off-time was 0 μs, the sputtering mode was equivalent to conventional DCMS, where the target ionization rate is limited. Upon extension of the pulse off-time, the peak power density applied on the target surface increased markedly, and the instantaneous energy released on the target rises considerably, resulting in a significant increase in the ionization rate of the target species. The ionized target species possess higher activity and react more easily with the reactive O atoms. Therefore, O content in the films deposited using HiPIMS mode was higher than that in the films deposited by DCMS mode. Nevertheless, the films’ composition remained almost unchanged and they were always oxygen-deficient, resulting in the formation of donor defects such as oxygen vacancies, thereby improving their conductivity.

**Figure 3:**
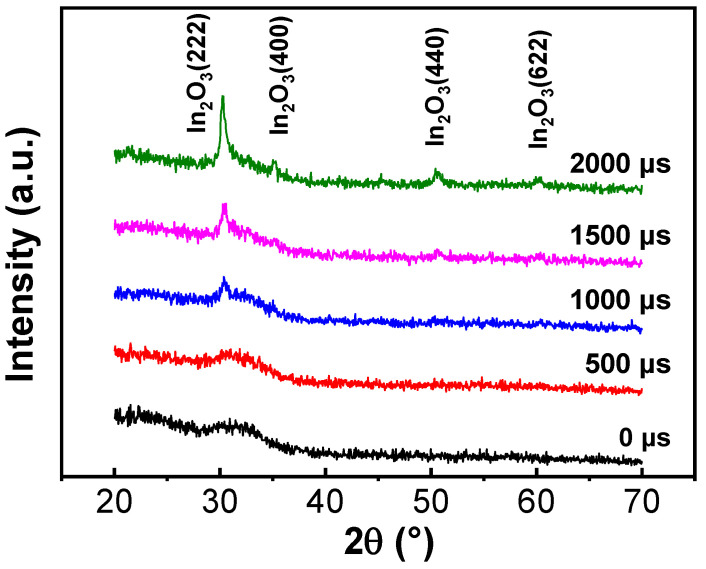
XRD patterns of ITZO films deposited at different pulse off-times.

**Figure 4:**
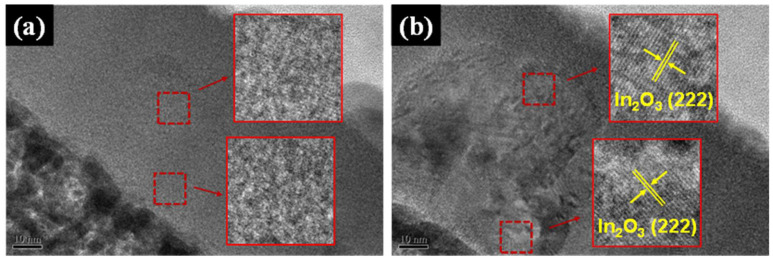
The cross-sectional TEM images as well as the corresponding enlarged images of ITZO films deposited with pulse off-times of (**a**) 0 μs and (**b**) 2000 μs.

Figure 3 shows the XRD spectra of the ITZO thin films deposited with various pulse off-times. Amorphous-like structures were obtained when the pulse off-times were 0 μs and 500 μs. No obvious diffraction peak could be detected in either of these films. As the pulse off-time extended to 1000 μs and 1500 μs, ITZO films began to crystallize and an In_2_O_3_ (222) diffraction peak emerged. Upon further extending the pulse off-time to 2000 μs, the films’ crystallinity increased significantly. Additional diffraction peaks of In_2_O_3_ (222), In_2_O_3_ (400), In_2_O_3_ (440), and In_2_O_3_ (622) were also identified. This behavior was related to higher instantaneous energy being bombarded on the target with the extension of pulse off-time; therefore, the sputtering species consequently possessed more kinetic energy. This promoted the nucleation and orderly growth of the ITZO films, thus improving the films’ crystallinity. The films’ crystalline features can be analyzed by TEM, as shown in Figure 4. The close-up lattice images in this figure are taken from the areas marked by red squares and produced through inverse Fourier transformation. In Figure 4a, many amorphous areas can be found in the ITZO film deposited with the pulse off-time of 0 μs. In contrast, In_2_O_3_ (222) planes with interplanar lattice spacing of about 2.9 Å can be clearly observed in Figure 4b, indicating that the films’ crystallinity increased with the extension of pulse off-time.

**Figure 5:**
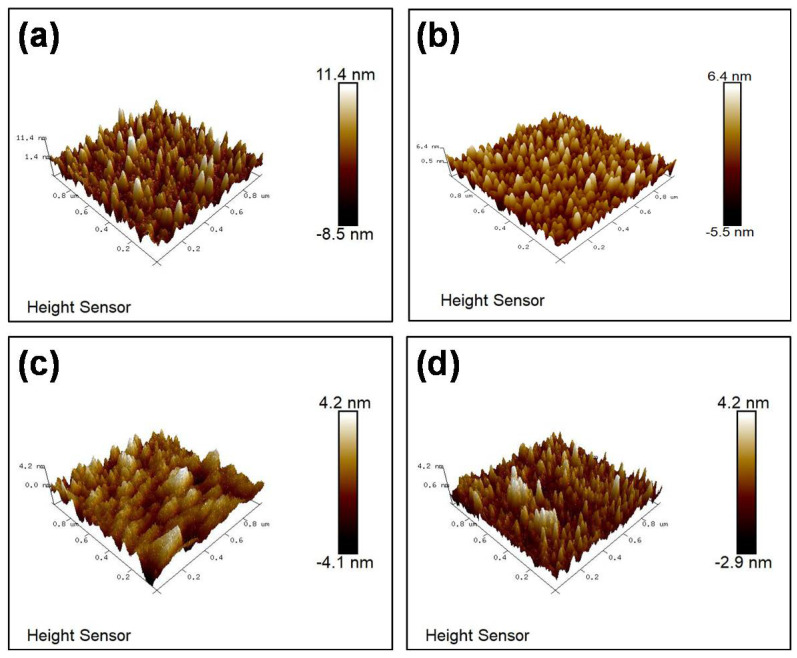
AFM images of ITZO films deposited with pulse off-times of (**a**) 0 μs, (**b**) 500 μs, (**c**) 1000 μs, and (**d**) 2000 μs.

The films’ surface morphology was characterized through AFM analysis (Figure 5). The roughness of the films decreased from 2.17 nm to 0.85 nm and further to 0.70 nm, as the pulse off-time extended from 0 μs to 1000 μs and on to 2000 μs. Due to the high-energy sputtering species bombardment of the substrate during the deposition process, the films became much denser and smoother under extended pulse off-time.

**Figure 6:**
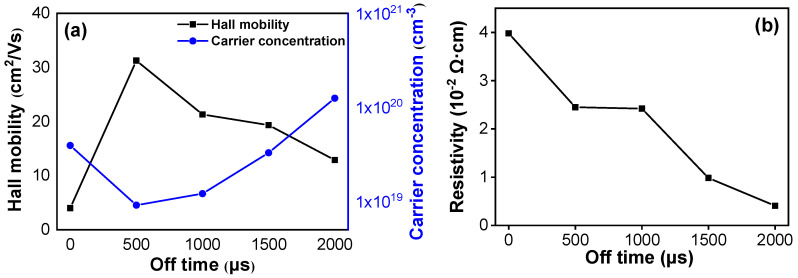
Electrical properties of ITZO films deposited at different pulse off-times: (**a**) the carrier mobility and carrier concentration and (**b**) the films’ resistivity.

The films’ electrical properties as analyzed by Hall measurement are shown in Figure 6. The variation of the carrier concentration and the carrier mobility are summarized in Figure 6a. As the pulse off-time was raised from 0 to 500 μs, the deposition mode changed from DC mode to HiPIMS mode. Due to the higher kinetic energy of the sputtering species bombarding the substrate during the HiPIMS deposition mode, a denser film with a low amount of defects was obtained. As a result, the carrier concentration decreased from 3.92 × 10^19^ cm^−3^ to 9.11 × 10^18^ cm^−3^; while the carrier scattering reduced, and the carrier mobility increased greatly from 3.99 cm^2^V^−1^s^−1^ to 31.25 cm^2^V^−1^s^−1^. Upon further extending the pulse off-time, the films changed from an isotropic amorphous structure to an anisotropic polycrystalline structure, with more grain boundaries introduced. This increased the probability of grain boundary scattering and hindered the carrier migration, resulting in a decrease in the carrier mobility. Similar behavior has also been found in nitrogen-doped ITZO films [29]. In addition, the target ionization rate enhanced with the extension of the pulse off-time, thereby raising the activity of the doping species during the sputtering process. Thus, the substitution of In^3+^ ions by Sn^4+^ occurred more readily. This substitution leads to lattice distortion and aids in the formation of V_O_ (oxygen vacancies) and Sn_In_ (the substitution of In^3+^ by Sn^4+^) donor defects, which both improve the carrier concentration.

The film’s resistivity is related to the carrier mobility and carrier concentration. Their relationship is calculated using the following equation [30]:

(1) ρ=1(e × μ × N)

where *ρ* is the film’s resistivity, *e* is the electron charge, *μ* is the carrier mobility, and *N* is the carrier concentration. Through the combined effects of carrier mobility and carrier concentration, the variation of the films’ resistivity as a function of pulse off-time is shown in Figure 6b. It decreased from 3.98 × 10^−2^ Ω·cm to 4.07 × 10^−3^ Ω·cm as the pulse off time rises from 0 μs to 2000 μs.

## 4. Conclusions

In this work, ITZO thin films were deposited on glass substrates at room temperature through HiPIMS technology with various pulse off-times. The microstructures and electrical properties of the films were investigated. The results show that compared with the ITZO film deposited under DCMS mode, ITZO films deposited using HiPIMS mode are denser and possess smoother surface morphology. As the pulse off-time was extended, the crystallinity of ITZO films enhanced, and the film’s resistivity effectively reduced. The optimal resistivity of about 4.07 × 10^−3^ Ω·cm was achieved when the pulse off-time was 2000 μs. This result indicates that through utilizing HiPIMS technology, ITZO films with controllable carrier concentration and carrier mobility in addition to controllable resistivity can be produced, which is desirable in the production of films for applications in various optoelectronic devices.

## Figures and Tables

**Table 1:** Sputtering parameters maintained during deposition of ITZO thin films.

Parameters	Value	Parameters	Value
Target	ITZO	Background pressure (Pa)	<7 × 10^−4^
Substrate	Glass/silicon	Working pressure (Pa)	0.7
*t*_on_ (µs)	50	Ar flow rate (sccm)	20
*t*_off_ (µs)	0 → 2000	Deposition temp. (°C)	ambient
Power (W)	300	Film thickness (nm)	~100

**Table 2:** The variation of duty cycle and peak power density as a function of *t*_off_ used in each experiment (*t*_on_ = 50 μs).

*t*_off_ (μs)	0	500	1000	1500	2000
Duty cycle (%)	100	9.09	4.72	3.23	2.44
Peak power density (W/cm^2^)	6.42	189.47	257.89	442.11	531.97

**Table 3:** Atomic concentration of ITZO films deposited at different pulse off-times.

*t*_off_ (μs)	In	Sn	Zn	O
0	54.69 (±0.1) at.%	3.49 (±0.1) at.%	1.19 (±0.1) at.%	40.63 (±0.1) at.%
500	52.95 (±0.1) at.%	3.47 (±0.1) at.%	1.03 (±0.1) at.%	42.56 (±0.1) at.%
1000	52.31 (±0.1) at.%	3.32 (±0.1) at.%	1.19 (±0.1) at.%	43.18 (±0.1) at.%
1500	52.91 (±0.1) at.%	3.56 (±0.1) at.%	1.03 (±0.1) at.%	42.50 (±0.1) at.%
2000	51.67 (±0.1) at.%	3.89 (±0.1) at.%	1.14 (±0.1) at.%	43.30 (±0.1) at.%

## References

[1] Lee C.C., Wang C.W. (2020). Interfacial fracture investigation of patterned active matrix OLED driven by amorphous-Si TFTs under film-type packaging technology. Appl. Surf. Sci..

[2] Hong Y., Wu M., Bae J.H., Hong S., Jeong Y., Jang D., Kim J.S., Hwang C.S., Park B.-G., Lee J.H. (2020). A new sensing mechanism of Si FET-based gas sensor using pre-bias. Sens. Actuators B Chem..

[3] Song J.H., Kim K.S., Mo Y.G., Choi R., Jeong J.K. (2014). Achieving High Field-Effect Mobility Exceeding 50 cm^2^/Vs in In-Zn-Sn-O Thin-Film Transistors. IEEE Electron Device Lett..

[4] Sun H., Chen S.C., Peng W.C., Wen C.K., Wang X., Chuang T.H. (2018). The influence of oxygen flow ratio on the optoelectronic properties of p-Type Ni_1−x_O films deposited by ion beam assisted sputtering. Coatings.

[5] Tiwari B., Bahubalindruni P.G., Santos A., Santa A., Figueiredo C., Pereira M., Martins R., Fortunato E., Barquinha P. (2020). Low-Voltage High-Speed Ring Oscillator With a-InGaZnO TFTs. IEEE J. Electron Devices Soc..

[6] Marrani A.G., Bonomo M., Dini D. (2019). Adsorption Dynamics of Redox Active Species onto Polarized Surfaces of Sensitized NiO. ACS Omega.

[7] Kim J., Park J., Yoon G., Khushabu A., Kim J.S., Pae S., Cho E.C., Yi J. (2020). Effect of IGZO thin films fabricated by Pulsed-DC and RF sputtering on TFT characteristics. Mater. Sci. Semicond. Process..

[8] Sun H., Jen S.U., Chiang H.P., Chen S.C., Lin M.H., Chen J.Y., Wang X. (2017). Investigation of optoelectronic performance in In, Ga co-doped ZnO thin films with various In and Ga levels. Thin Solid Films.

[9] Chae M.S., Park J.H., Son H.W., Hwang K.S., Kim T.G. (2018). IGZO-based electrolyte-gated field-effect transistor for in situ biological sensing platform. Sens. Actuators B Chem..

[10] Jia J., Torigoshi Y., Shigesato Y. (2013). In situ analyses on negative ions in the indium-gallium-zinc oxide sputtering process. Appl. Phys. Lett..

[11] Tomai S., Nishimura M., Itose M., Matuura M., Kasami M., Matsuzaki S., Kawashima H., Utsumo F., Uano K. (2012). High-Performance Thin Film Transistor with Amorphous In_2_O_3_–SnO_2_–ZnO Channel Layer. Jpn. J. Appl. Phys..

[12] Jang J., Kim D.G., Kim D.M., Choi S.J., Lim J.H., Lee J.H., Kim Y.S., Ahn B.D., Kim D.H. (2014). Investigation on the negative bias illumination stress-induced instability of amorphous indium-tin-zinc-oxide thin film transistors. Appl. Phys. Lett..

[13] Jia J., Torigoshi Y., Kawashima E., Utsuno F., Yano K., Shigesato Y. (2015). Amorphous indium-tin-zinc oxide films deposited by magnetron sputtering with various reactive gases: Spatial distribution of thin film transistor performance. Appl. Phys. Lett..

[14] Noh J.Y., Kim H., Nahm H.H., Kim Y.S., Kim D.H., Ahn B.D., Lim J.H., Kim G.H., Lee J.H., Song J. (2013). Cation composition effects on electronic structures of In-Sn-Zn-O amorphous semiconductors. J. Appl. Phys..

[15] Li Z.Y., Chen S.C., Huo Q.H., Liao M.H., Dai M.J., Lin S.S., Yang T.L., Sun H. (2019). Influence of sputtering power on the electrical properties of In-Sn-Zn oxide thin films deposited by high power impulse magnetron sputtering. Coatings.

[16] Wen L., Sahu B.B., Kim H.R., Han J.G. (2019). Study on the electrical, optical, structural, and morphological properties of highly transparent and conductive AZO thin films prepared near room temperature. Appl. Surf. Sci..

[17] Ayaz S., Mishra P.K., Sharma R.K., Kamal S., Sen S. (2020). Structural, Optoelectronic, and Electrochemical Properties of Zn_1–x_(Ga_0.5_Al_0.5_)_x_O Nanoparticles for Supercapacitor Applications. ACS Appl. Nano Mater..

[18] Li Z.Y., Yang H.Z., Chen S.C., Lu Y.B., Xin Y.Q., Yang T.L., Sun H. (2018). Impact of active layer thickness of nitrogen-doped In–Sn–Zn–O films on materials and thin film transistor performances. J. Phys..

[19] Cui Y., Li C.J., Li J., Xiong L.Y., Liu S. (2020). Characterization of FeCeAlY thin film deposited by magnetron sputtering and its corrosion resistance under high-temperature water vapor environment. Surf. Technol..

[20] Song S., Sun H., Chen S.C., Dai M., Wang K., Zheng X., Lu Y.B., Yang T.L., Yue Z.M. (2019). The adhesion strength and mechanical properties of SiC films deposited on SiAlON buffer layer by magnetron sputtering. Surf. Coat. Technol..

[21] Fan X., Huai X., Wang J., Jing L.C., Wang T., Liu J., Geng H.Z. (2021). Low surface roughness graphene oxide film reduced with aluminum film deposited by magnetron sputtering. Nanomaterials.

[22] Chen S.C., Kuo T.Y., Lin H.C., Chen R.Z., Sun H. (2020). Optoelectronic properties of p-type NiO films deposited by direct current magnetron sputtering versus high power impulse magnetron sputtering. Appl. Surf. Sci..

[23] Lin S.S., Gao D., Su Y.F., Xu W., Guo C.Q., Li H., Shi Q., Wei C.B., Dai M.J., Yang J.C. (2020). Effect of bias voltage on structure and properties of DLC films deposited by high power pulse magnetron sputtering. Mater. Res. Appl..

[24] Sun H., Kuo T.Y., Chen S.C., Chen Y.H., Lin H.C., Yazdi M.A.P., Billard A. (2019). Contribution of enhanced ionization to the optoelectronic properties of p-type NiO films deposited by high power impulse magnetron sputtering. J. Eur. Ceram. Soc..

[25] Ghailane A., Larhlimi H., Tamraoui Y., Makha M., Busch H., Fischer C.B., Alami J. (2020). The effect of magnetic field configuration on structural and mechanical properties of TiN coatings deposited by HiPIMS and dcMS. Surf. Coat. Technol..

[26] Chuang T.H., Wen C.K., Chen S.C., Liao M.H., Liu F., Sun H. (2020). p-type semi-transparent conductive NiO films with high deposition rate produced by superimposed high power impulse magnetron sputtering. Ceram. Int..

[27] Wang Z., Li Q., Yuan Y., Yang L., Zhang H., Liu Z., Ouyang J.T., Chen Q. (2019). The semi-conductor of ZnO deposited in reactive HiPIMS. Appl. Surf. Sci..

[28] Stranak V., Bogdanowicz R., Sezemsky P., Wulff H., Kruth A., Smietana M., Kratochvil J., Cada M., Hubicka Z. (2018). Towards high quality ITO coatings: The impact of nitrogen admixture in HiPIMS discharges. Surf. Coat. Technol..

[29] Jia J., Torigoshi Y., Suko A., Nakamura S.I., Kawashima E., Utsuno F., Shigesato Y. (2017). Effect of nitrogen addition on the structural, electrical, and optical properties of In-Sn-Zn oxide thin films. Appl. Surf. Sci..

[30] Chen S.C., Huang S.Y., Sakalley S., Paliwal A., Chen Y.H., Liao M.H., Sun H., Biring S. (2019). Optoelectronic properties of Cu_3_N thin films deposited by reactive magnetron sputtering and its diode rectification characteristics. J. Alloys Compd..

